# Supplementary material for: Laser-equipped gas reaction chamber for probing environmentally sensitive materials at near atomic scale
Source: PLoS One. 2022 Feb 9;17(2):e0262543. doi: 10.1371/journal.pone.0262543 (PMC8827481; doi:10.1371/journal.pone.0262543)
Supplement: S1 Fig — (PDF) [file pone.0262543.s001.pdf]

Fig S1 shows the calibrations performed on the half-grid in vacuum under two different temperature conditions. For vacuum, Figs S1(a) and S1(b) show the curves for the stage at room temperature (19°C) and at the minimum achievable cryogenic temperature (45K) respectively. Both seem to exhibit a linear relationship, displayed in a "y=mx+c" form. By itself, differences in stage temperature do not seem to affect pyrometer readings to a significant degree that we talk about in the paper. Our next supplementary figure exhibits this also.

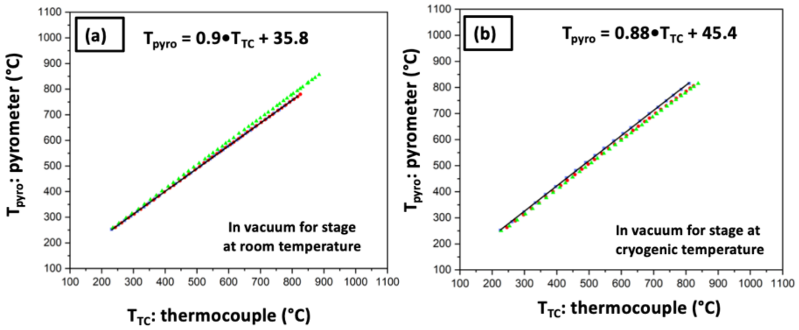

Fig S1. Calibration curves for 304SS grid in vacuum *(a) Stage at room temperature; and (b) stage at cryogenic temperature.*
