# Supplementary material for: Laser-equipped gas reaction chamber for probing environmentally sensitive materials at near atomic scale
Source: PLoS One. 2022 Feb 9;17(2):e0262543. doi: 10.1371/journal.pone.0262543 (PMC8827481; doi:10.1371/journal.pone.0262543)
Supplement: S2 Fig — Calibration curves for a high Mn steel grade for the candle geometry under different conditions. (PDF) [file pone.0262543.s002.pdf]

Fig S2 depicts the calibrations on different steel which is fairly resilient against change of conditions. The sample was fashioned into a candle geometry.

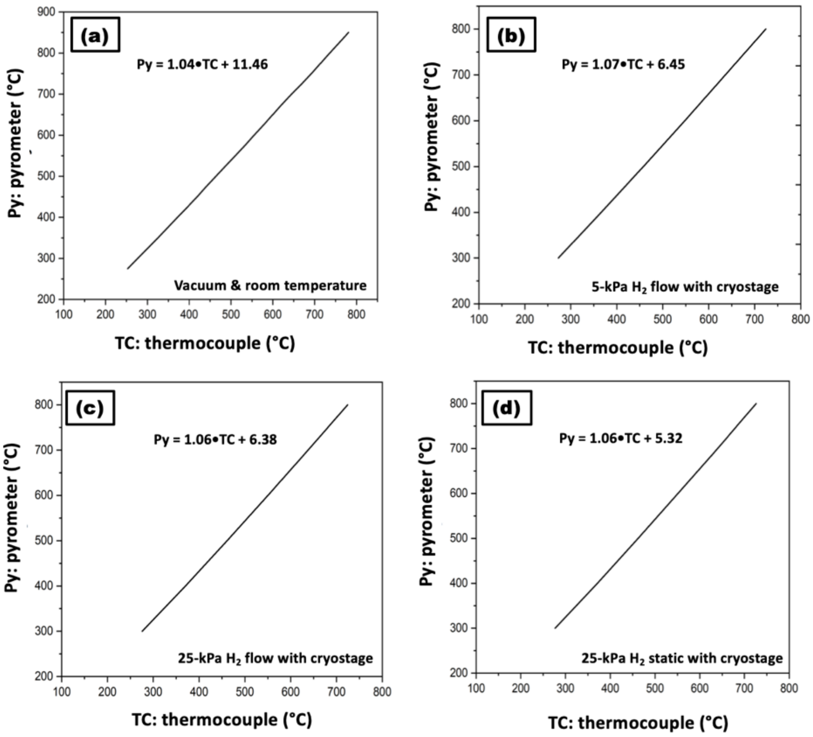

Fig S2. Calibration for candle geometry. *Calibration curves for a high Mn steel grade for the candle geometry under different conditions.*
