# Supplementary material for: Laser-equipped gas reaction chamber for probing environmentally sensitive materials at near atomic scale
Source: PLoS One. 2022 Feb 9;17(2):e0262543. doi: 10.1371/journal.pone.0262543 (PMC8827481; doi:10.1371/journal.pone.0262543)
Supplement: S3 Fig — Quenching curve depicting the quenching rate of at least 900°C/s in the first second. (PDF) [file pone.0262543.s003.pdf]

Figure S3 demonstrated that, with an active cryostage, a swift quenching rate of at least 900 °C.s$^{-1}$ in the first second was achieved. There is definitely a study to be made in making further investigations in calibrating laser heating, but our work revealing that it would have to be so for every particular application, so the study of this was determined beyond the scope of this manuscript. This will be revisited again when we continue our work on developing different targets and investigate carbon monoxide, oxygen and nitrogen atmospheres.

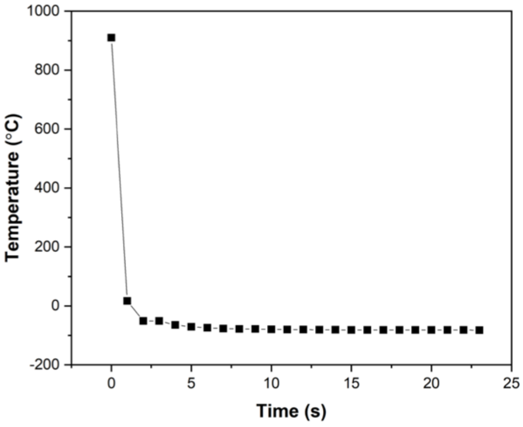


***Fig S3. Quenching speed.*** *Quenching curve depicting the quenching rate of at least 900 °C/s in the first second.*
