# Supplementary material for: Laser-equipped gas reaction chamber for probing environmentally sensitive materials at near atomic scale
Source: PLoS One. 2022 Feb 9;17(2):e0262543. doi: 10.1371/journal.pone.0262543 (PMC8827481; doi:10.1371/journal.pone.0262543)
Supplement: S4 Fig — Focused ion beam liftout protocol for atom probe samples (a-c) Site specific liftout procedure for preparing APT tip for D gas charging on the high manganese TWIP steel. (a) A lamella from a sample is cut. (b) The lamella was then lifted out using a micromanipulator and is (c) welded to the mounting grid. (d) The sample is then milled down to appropriate atom probe sample dimensions (<100-nm end radius). (PDF) [file pone.0262543.s004.pdf]

Lift-out with Focused ion beam for Reacthub applications

The procedure as shown by Fig S4, starting from protecting the region of interest (ROI) with electron-beam- and ion-beam-deposited Pt capping layers and making trenches each side (Fig S4(a)), lifting out the made lamella (Fig S4(b)), mounting a part of the lamella upon one of the three prepared conical mounts on a pre-processed Reacthub grid (Fig S4(c)), and finally milled down to an appropriate shape for an atom probe specimen (Fig S4(d)).

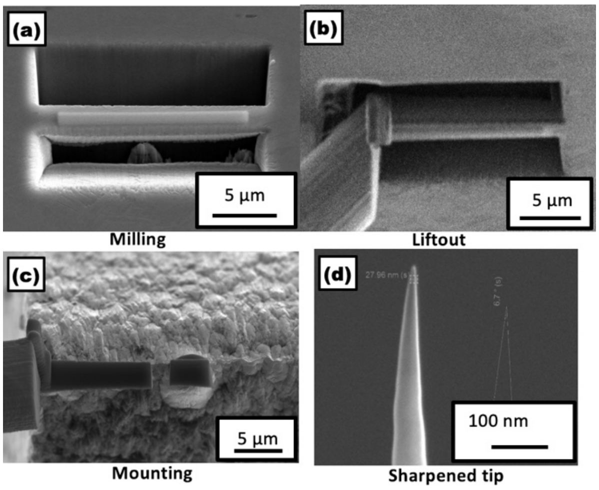

Fig S4. Focused ion beam liftout protocol for atom probe samples *(a-c) Site specific liftout procedure for preparing APT tip for D gas charging on the high manganese TWIP steel. (a) A lamella from a sample is cut. (b) The lamella was then lifted out using a micromanipulator and is (c) welded to the mounting grid. (d) The sample is then milled down to appropriate atom probe sample dimensions (<100-nm end radius).*
