# Supplementary material for: Laser-equipped gas reaction chamber for probing environmentally sensitive materials at near atomic scale
Source: PLoS One. 2022 Feb 9;17(2):e0262543. doi: 10.1371/journal.pone.0262543 (PMC8827481; doi:10.1371/journal.pone.0262543)
Supplement: S1 Text — (DOCX) [file pone.0262543.s005.docx]

**SUPPORTING INFORMATION**

**Additional laser heating calibrations**

Figure A shows the calibrations performed on the half-grid in vacuum under two different temperature conditions. For vacuum, Figures A(a) and A(b) show the curves for the stage at room temperature (19°C) and at the minimum achievable cryogenic temperature (45K) respectively. Both seem to exhibit a linear relationship, displayed in a “y=mx+c” form. By itself, differences in stage temperature do not seem to affect pyrometer readings to a significant degree that we talk about in the paper. Our next supplementary figure exhibits this also.

*
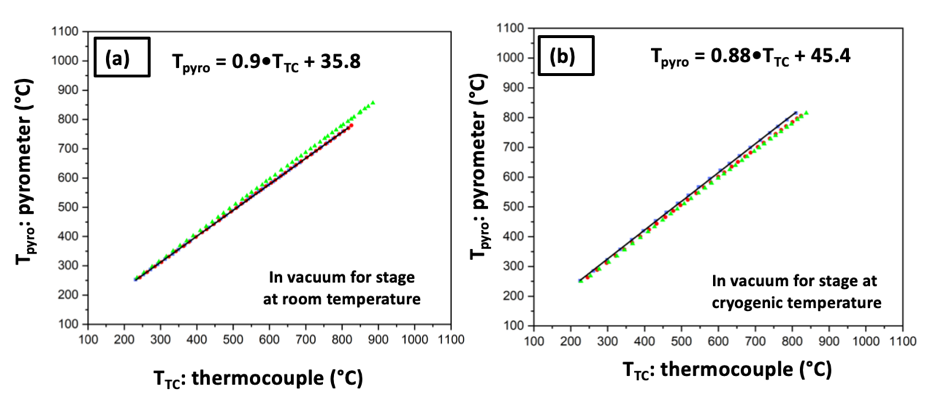
*

***Fig A.*** ***Calibration curves for 304SS grid in vacuum*** *(a) Stage at room temperature; and (b) stage at cryogenic temperature.*

Figure B depicts the calibrations on different steel which is fairly resilient against change of conditions. The sample was fashioned into a candle geometry.


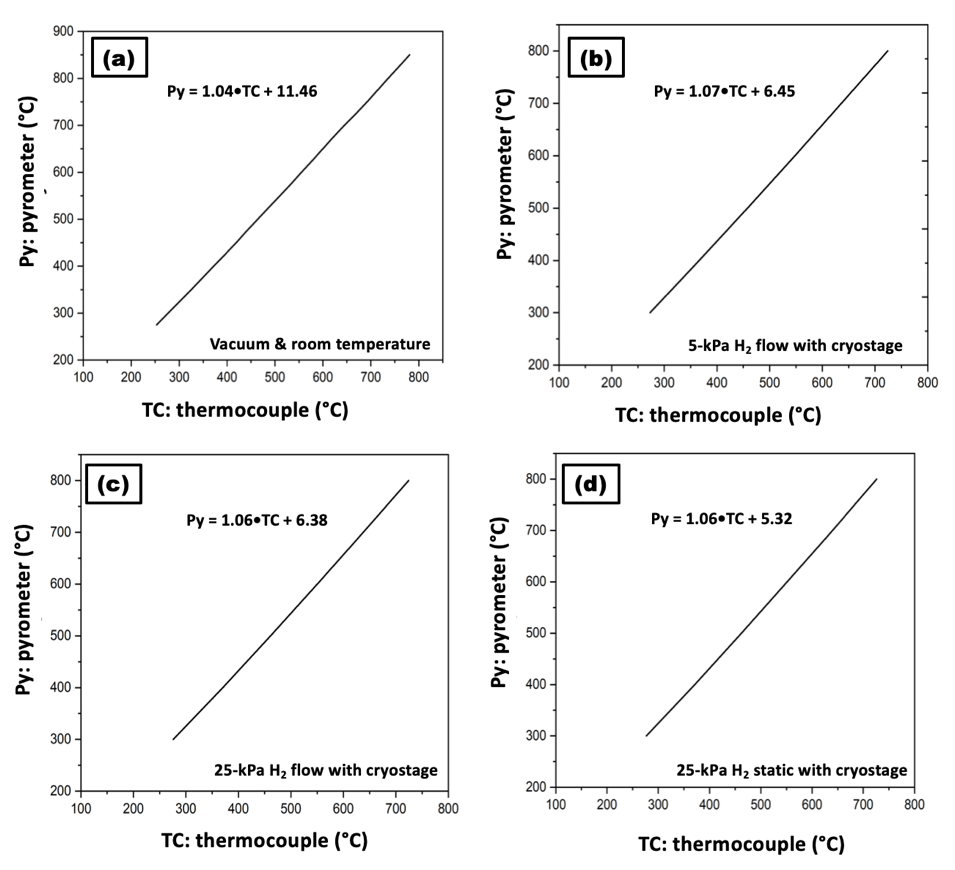


***Fig B.*** ***Calibration for candle geometry.*** *Calibration curves for a high Mn steel grade for the candle geometry under different conditions.*

Figure C demonstrated that, with an active cryostage, a swift quenching rate of at least 900 °C.s^-1^ in the first second was achieved. There is definitely a study to be made in making further investigations in calibrating laser heating, but our work revealing that it would have to be so for every particular application, so the study of this was determined beyond the scope of this manuscript. This will be revisited again when we continue our work on developing different targets and investigate carbon monoxide, oxygen and nitrogen atmospheres.


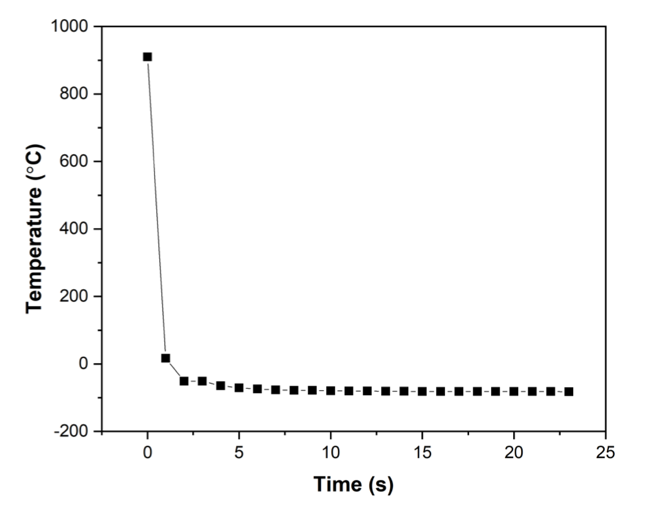


***Fig C.*** ***Quenching speed.*** *Quenching curve depicting the quenching rate of at least 900 °C/s in the first second.*

**Lift-out with Focused ion beam for Reacthub applications**

The procedure as shown by Figure D, starting from protecting the region of interest (ROI) with electron-beam- and ion-beam-deposited Pt capping layers and making trenches each side (Figure D(a)), lifting out the made lamella (Figure D(b)), mounting a part of the lamella upon one of the three prepared conical mounts on a pre-processed Reacthub grid (Figure D(c)), and finally milled down to an appropriate shape for an atom probe specimen (Figure D(d)).


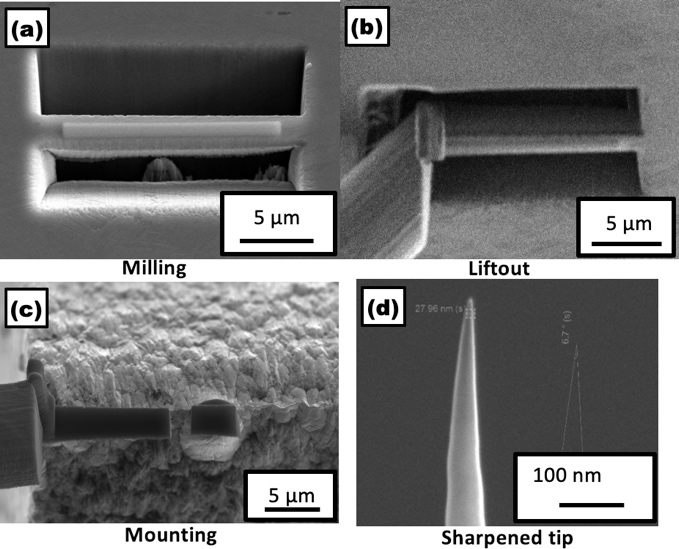


***Fig D. Focused ion beam liftout protocol for atom probe samples*** *(a-c) Site specific liftout procedure for preparing APT tip for D gas charging on the high manganese TWIP steel. (a) A lamella from a sample is cut. (b) The lamella was then lifted out using a micromanipulator and is (c) welded to the mounting grid. (d) The sample is then milled down to appropriate atom probe sample dimensions (<100-nm end radius).*
