# Supplementary material for: Laser-equipped gas reaction chamber for probing environmentally sensitive materials at near atomic scale
Source: PLoS One. 2022 Feb 9;17(2):e0262543. doi: 10.1371/journal.pone.0262543 (PMC8827481; doi:10.1371/journal.pone.0262543)
Supplement: Attachment — Submitted filename: comment-2021-11-11-PLOS ONE.docx [file pone.0262543.s006.docx]

In this paper, a new thermochemical reaction chamber named the "Reacthub" is designed and established to study the effect of thermochemical treatments on materials in specific gaseous atmospheres, which also allows for transferring treated materials under cryogenic & ultrahigh vacuum (UHV) workflow conditions for characterisation by either atom probe or scanning Xe+/electron microscopes. This work would contribute to the quantitative atomic-scale characterizations of environmentally sensitive materials and their transient states. However, the topic addressed in this manuscript is not within the scope of **PLOS ONE**. Therefore, I personally do not recommend its publication in this journal of “**PLOS ONE**”.
