# Supplementary material for: Laser-equipped gas reaction chamber for probing environmentally sensitive materials at near atomic scale
Source: PLoS One. 2022 Feb 9;17(2):e0262543. doi: 10.1371/journal.pone.0262543 (PMC8827481; doi:10.1371/journal.pone.0262543)
Supplement: Attachment — Submitted filename: Response to Reviewers.docx [file pone.0262543.s007.docx]

Dear PLOSone editor & reviewers,

Thank you for your careful consideration of our research article entitled “*Laser-equipped gas reaction chamber for probing environmentally sensitive materials at near atomic scale”.*

Reviewer 1’s recommendation that the manuscript be refused on the basis that it is beyond PLOSone’s scope is no doubt made under the misapprehension that PLOSone is specifically a journal for the biological or medical sciences. A “big tent” philosophy admitting many diverse fields has allowed this journal to remain competitive. So we thank Reviewer 1 for their expressed concern but find it unjustified.

Reviewer 2 posed two questions prompting the small changes below.

*1. What was the effort needed to construct this custom made stage? For instance did the construction of the reaction stage involve much custom made machining ?*

**Answered by inserting in Section 4a**: The stage component was custom-made to fit the cryogenic head but in all other respects was engineered along standard designs so as to fit Cameca’s atom probe specimen pucks.

*2. Does the laser / cryo combination enable one to control the rate of temperature change of the sample?*

**Answered by inserting in Section 4c:** The laser’s control software permits increasing and decreasing controlled ramps from one temperature to another over a specified period, but this capability was not demonstrated here.

We have uploaded the required versions, one showing changes and one without mark-up.

Kind regards,

Dr Leigh Stephenson
